# SERPINH1 regulates EMT and gastric cancer metastasis via the Wnt/β-catenin signaling pathway

**DOI:** 10.18632/aging.102831

**Published:** 2020-02-24

**Authors:** Shan Tian, Pailan Peng, Jiao Li, Huan Deng, Na Zhan, Zhi Zeng, Weiguo Dong

**Affiliations:** 1 Department of Gastroenterology, Renmin Hospital of Wuhan University, Wuhan 430061, Hubei Province, China; 2 Department of Gastroenterology, The Affiliated Hospital of Guizhou Medical University, Guiyang 550004, Guizhou Province, China; 3 Department of Pathology, Renmin Hospital of Wuhan University, Wuhan 430061, Hubei Province, China

**Keywords:** epithelial-mesenchymal transition, SERPINH1, gastric cancer, Wnt/β-catenin pathway, therapeutic target

## Abstract

In this study, we investigated the role of SERPINH1 in gastric cancer (GC) progression. GC patient tissues show significantly higher SERPINH1 mRNA and protein levels than normal gastric mucosal tissues. GC patients with high SERPINH1 expression are associated with lymph node metastasis and poor prognosis. SERPINH1 mRNA levels negatively correlate with E-cadherin mRNA levels and positively correlate with levels of N-cadherin, MMP2, and MMP9 mRNA levels. This suggests SERPINH1 regulates epithelial to mesenchymal transition (EMT). SERPINH1 expression was significantly higher in the HGC-27, AGS, MGC-803, and SGC-7901 GC cell lines than in the GES-1 normal gastric mucosal cell line. In SERPINH1-silenced SGC-7901 cells, survival, colony formation, migration and invasion were all reduced, whereas they were all enhanced in SERPINH1-overexpressing MGC-803 cells. Levels of WNT/β-catenin signaling pathway proteins, including β-catenin, Wnt2, GSK-3β, p-GSK-3β, NF-κB P65, Snail1, Slug and TWIST, were all reduced in SERPINH1-silenced SGC-7901 cells, and increased in the SERPINH1-overexpressing MGC-803 cells. Inhibition of SERPINH1 protein using Co1003 significantly decreased survival, invasion, and migration of GC cells. SERPINH1 thus appears to regulate EMT and GC progression via the Wnt/β-catenin pathway, making SERPINH1 a potential prognostic biomarker and therapeutic target in GC patients.

## INTRODUCTION

Gastric cancer (GC) is the third most common cause of cancer-related deaths in the world, and the most common gastrointestinal malignancy in China [1–3]. Despite great advances in surgery and adjuvant treatments, the 5-year survival rate of GC patients is poor because of cancer recurrence due to metastases [4]. Hence, elucidating the molecular mechanisms involved in GC metastasis is necessary to improve survival outcomes [5].

Metastasis is a complex process that involves epithelial-mesenchymal transition (EMT) of gastric cancer cells, in which cancer cells acquire mesenchymal properties that enable them to access the blood stream and invade other organs of the human body [6–9]. EMT involves cellular reprogramming through changes in gene transcription [10]. Hence, understanding the molecular mechanisms underlying EMT is required to identify newer strategies to treat GC.

The Serpin Family H Member 1 (*SERPINH1*) gene encodes a protein called HSP47, which is required for the correct folding and secretion of collagen [11]. SERPINH1 is aberrantly expressed in cervical cancer [12], breast cancer [13], glioblastoma [14] and colorectal cancer [15]. SERPINH1 promotes invasion and metastasis of breast cancer cells by regulating the expression of several extracellular matrix (ECM) proteins [13]. In our present work, gene enrichment analysis shows that genes co-expressed with SERPINH1 in GC are mainly involved in the EMT pathway. Besides, Wnt/β-catenin signaling pathway regulates EMT in gastric cancer [16]. However, it is not clear if SERPINH1 regulates EMT via Wnt/β-catenin pathway in GC is not clear.

In this study, we investigated the prognostic significance of SERPINH1 by analyzing GC patient tissues. Furthermore, we analyzed the relationship between SERPINH1, EMT and Wnt/β-catenin pathway using SERPINH1 knockdown and overexpressing GC cell lines. Finally, we analyzed the anti-tumor effects of Col003, a small molecule inhibitor of the SERPINH1 protein.

## RESULTS

### SERPINH1 mRNA levels are significantly upregulated in GC tissues

**Figure 1:**
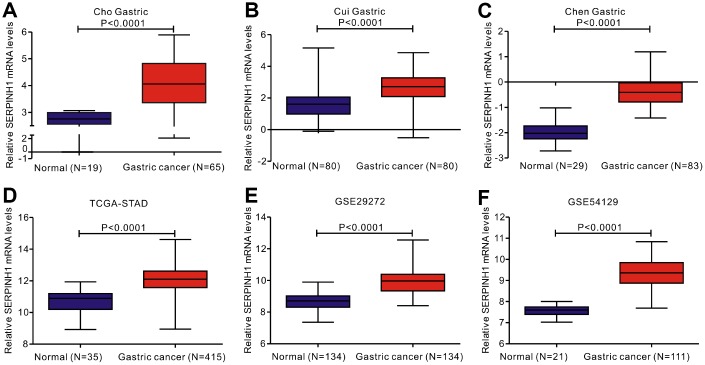
**Analysis of SERPINH1 mRNA expression in normal and gastric cancer (GC) tissues from 3 public databases.** SERPINH1 mRNA levels are significantly lower (P<0.0001) in normal gastric mucosal than that in gastric cancer tissue samples in the (**A**) Cho (Normal=19; Tumor=65), (**B**) Cui (Normal=80; Tumor=80), and (**C**) Chen (Normal=29; Tumor=83) Gastric datasets from the Oncomine database; (**D**) STAD dataset (Normal=35; Tumor=415) from the TCGA database; and (**E**) GSE29272 (Normal=134; Tumor=134) and (**F**) GSE54129 (Normal=21; Tumor=111) datasets from the GEO databases.

**Figure 2:**
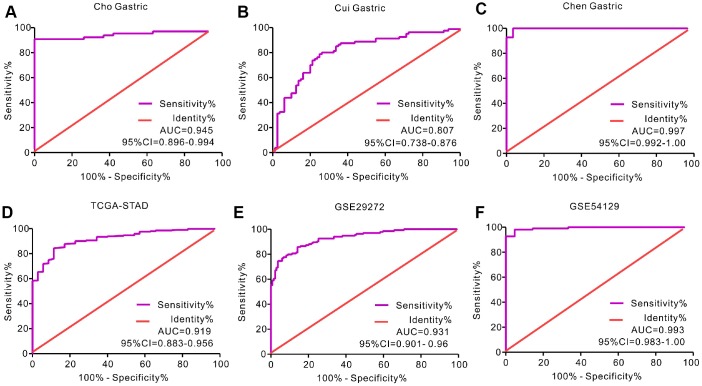
**Receiver operating characteristic (ROC) curve analysis to determine diagnostic relevance of SERPINH1 mRNA levels in GC patients.** ROC curve analysis of SERPINH1 mRNA levels in the (**A**) Cho (AUC=0.945), (**B**) Cui (AUC=0.807), and (**C**) Chen (AUC=0.997) Gastric datasets from the Oncomine database; (**D**) STAD dataset (AUC=0.919) from the TCGA database; and (**E**) GSE29272 (AUC=0.931) and (**F**) GSE54129 (AUC=0.993) datasets from the GEO databases.

We analyzed gene expression data in the Oncomine (Cho Gastric, Cui Gastric, and Chen Gastric datasets), The Cancer Genome Atlas (TCGA), and Gene expression omnibus (GEO; GSE29272 and GSE54129 datasets) databases to compare SERPINH1 mRNA levels in the tumor and normal gastric mucosal tissues from GC patients (N=888). The GC tissues showed significantly higher SERPINH1 mRNA expression than the adjacent tissues (N=318; Figure 1). Next, we performed ROC curve analysis and found that the SERPINH1 mRNA levels were of significant diagnostic value in all six datasets (Figure 2). We obtained the highest diagnostic value for the SERPINH1 mRNA in the Chen Gastric dataset (AUC=0.997, 95%CI=0.992-1.00) by using a cut-off value of -1.435 (Table 1). Furthermore, correlation analysis using the TCGA-STAD dataset showed that GC patients with high SERPINH1 mRNA levels showed poorer OS rates than those with lower SERPINH1 mRNA levels. Moreover, Kaplan-Meier survival curve analysis of GC patients from the TCGA database (N=388) showed that GC patients with high SERPINH1 mRNA expression were associated with poorer OS (HR=1.49, 95%CI=1.065-2.086, P=0.0198, Figure 4A) and RFS (HR=1.89, 95%CI=1.32-3.158, P=0.015, Figure 4B) than GC patients with low SERPINH1 mRNA expression. We confirmed the prognostic significance of SERPINH1 by analyzing GC patients from the GEO datasets using the Kaplan Meier-plotter database [17]. The results showed that GC patients with high SERPINH1 mRNA levels were associated with poorer OS (HR=1.56, 95%CI=1.31-1.85, P<0.0001, Figure 4C) and PFS (HR=1.73, 95%CI=1.41-2.12, P<0.0001, Figure 4D) than the GC patients with low SERPINH1 expression.

**Figure 3:**
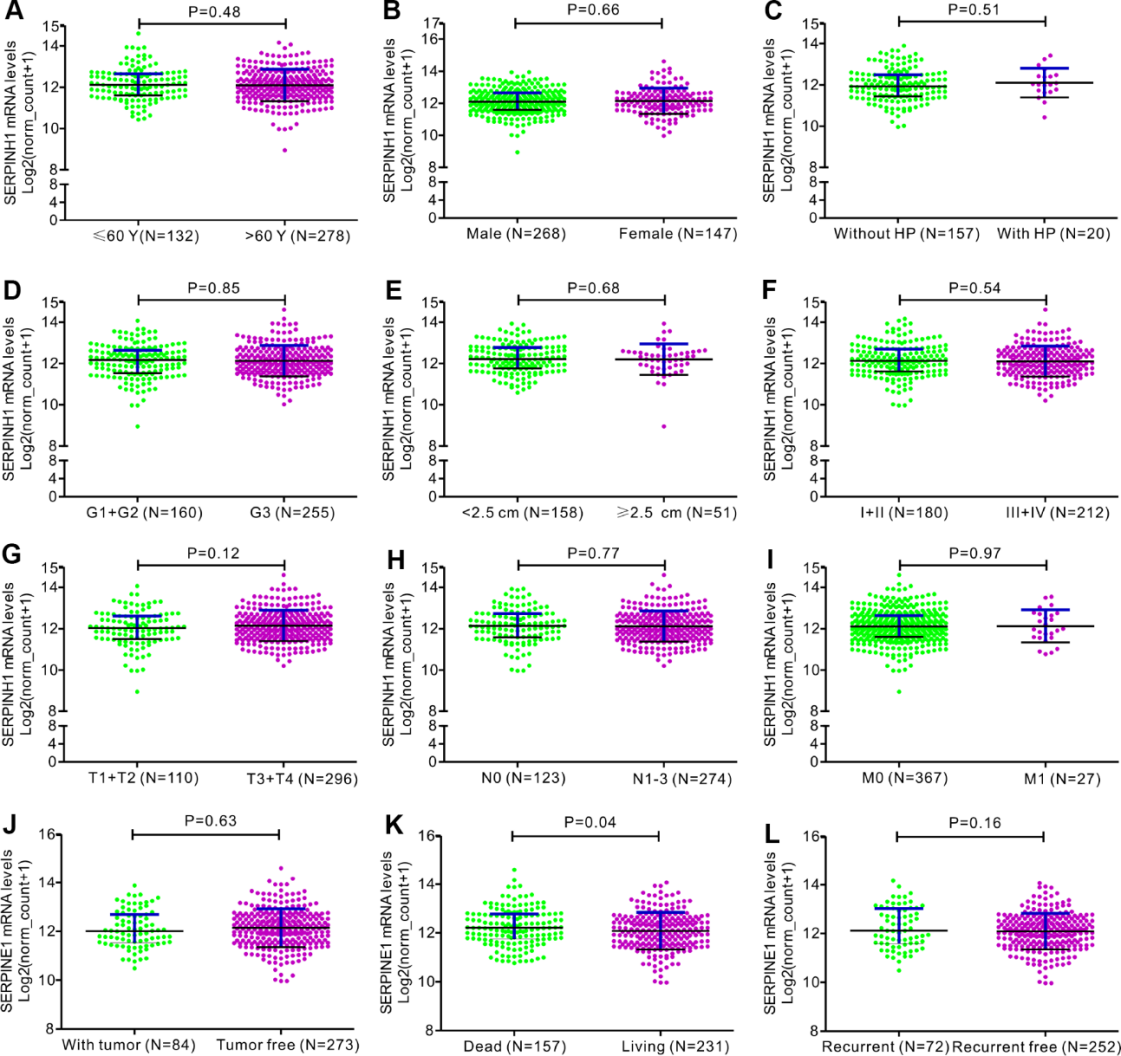
**Correlation analyses between SERPINH1 mRNA levels and different clinicopathological characteristics of GC patients.** The association between SERPINH1 levels and clinicopathological characteristics of GC patients, including (**A**) Age (P=0.48); (**B**) Gender (P=0.66); (**C**) Infection of *Helicobacter pylori* (HP; P=0.51); (**D**) Tumor grade (G) stage (P=0.85); (**E**) Tumor size (P=0.68); (**F**) Tumor Node Metastasis (TNM) stage (P=0.54); (**G**) Tumor (T) stage (P=0.12); (**H**) Node (N) stage (P=0.77); (**I**) Metastasis (M) stage (P=0.97); (**J**) Tumor status (P=0.63); (**K**) Overall Survival (OS; P=0.04); (**L**) Relapse-free survival (RFS; P=0.16).

**Table 1:** The diagnostic value of SERPINH1 mRNA for GC from six datasets.

**Dataset**	**AUC**	**95% CI**	**P value**	**Cut-off value**	**Sensitivity**	**Specificity**
Cho Gastric	0.945	0.899-0.994	<0.0001	3.068	90.77%	100.0%
Cui Gastric	0.807	0.738- 0.876	<0.0001	1.952	80.0%	73.75%
Chen Gastric	0.997	0.992-1.00	<0.0001	-1.435	100%	96.55%
TCGA-STAD	0.919	0.883- 0.956	<0.0001	11.4	84.34%	88.57%
GSE29272	0.931	0.901- 0.960	<0.0001	9.135	86.57%	84.33%
GSE54129	0.993	0.983-1.00	<0.0001	7.816	98.20%	95.24%

**Figure 4:**
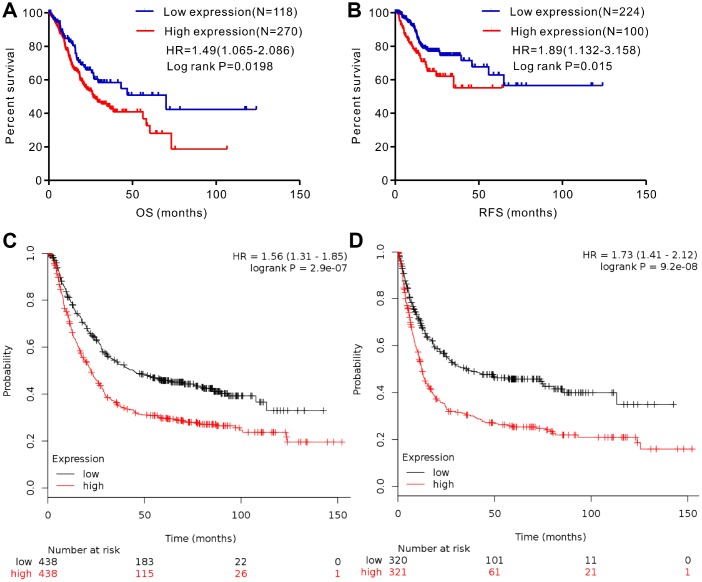
**Analysis of the prognostic significance of SERPINH1 mRNA levels in GC patients.** (**A**) Patients with high SERPINH1 mRNA levels show significantly poorer OS than patients with low SERPINH1 mRNA levels in the TCGA-STAD dataset (N=388, HR=1.49, P=0.0198). (**B**) Patients with high SERPINH1 mRNA levels show poorer RFS than patients with low SERPINH1 mRNA levels in the TCGA-STAD dataset (N=324, HR=1.89, P=0.015). (**C**) GC patients with high SERPINH1 mRNA levels show poorer OS than the GC patients with low SERPINH1 levels in the Kaplan-Meier Plotter database (N=876, HR=1,56, P<0.0001). (**D**) GC patients with high SERPINH1 mRNA levels show poorer PFS than GC patients with low SERPINH1 levels in the Kaplan-Meier Plotter database (N=641, HR=1.73, P<0.0001).

### SERPINH1 protein expression is upregulated in GC tissues

**Figure 5:**
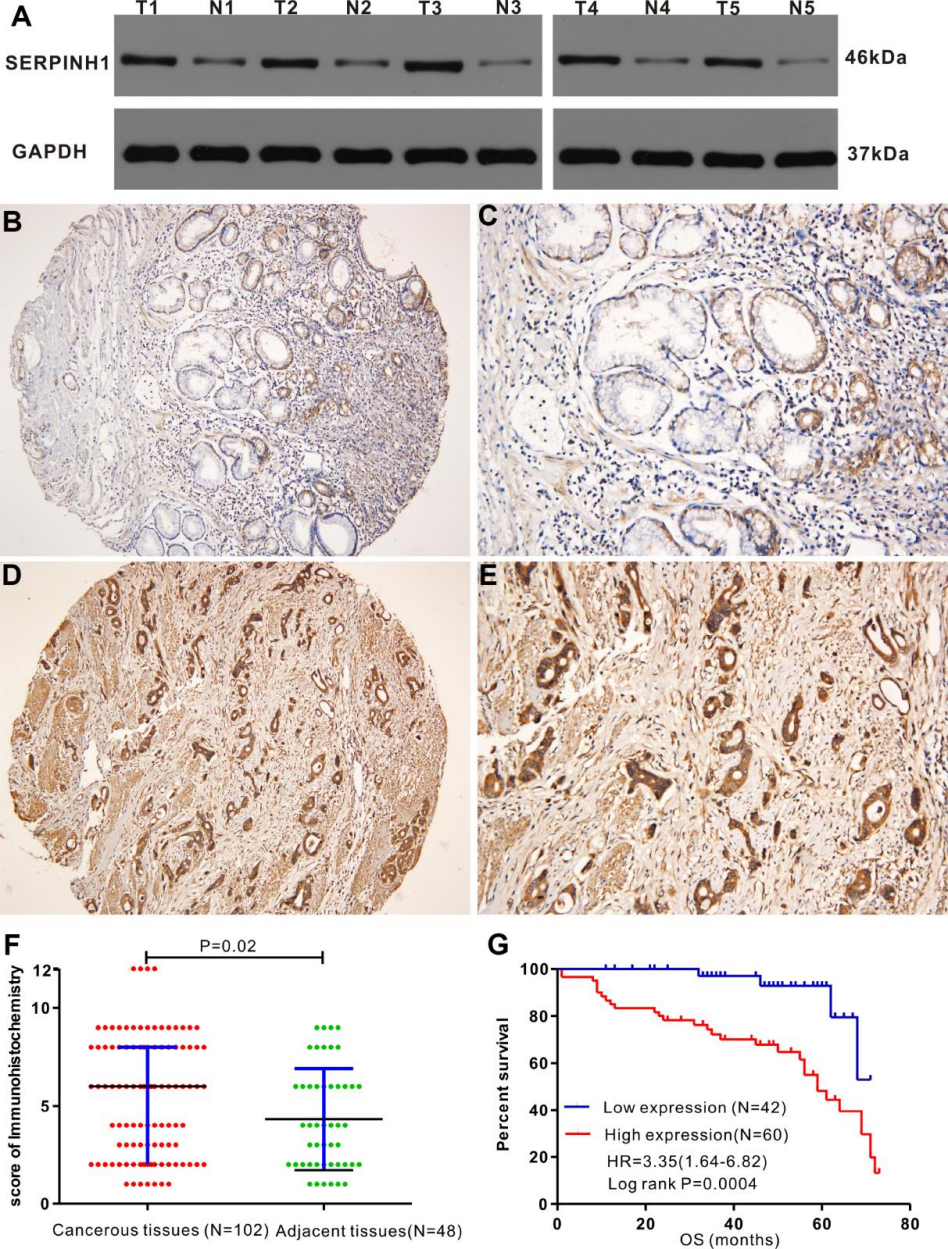
**Immunohistochemical analysis of SERPINH1 protein expression in human GC tissues.** (**A**) Immunohistochemical (IHC) analysis shows that SERPINH1 protein levels are significantly higher in five pairs of matched GC tissues compared with the adjacent non-tumor gastric mucosal tissues. (**B**–**E**) Representative images show IHC staining of SERPINH1 protein in (**B**, **C**) normal gastric mucosal tissues and (**D**, **E**) gastric cancer tissues at 100X and 200X magnification, respectively. (**F**) Comparison of IHC scores show that SERPINH1 protein expression is significantly higher (P=0.02) in gastric cancer tissues (N=102) compared with adjacent non-tumor gastric tissues (N=48). (**G**) Survival curve analysis shows that GC patients with high SERPINH1 protein levels exhibit poorer OS than patients with low SERPINH1 protein levels (HR=3.35, P=0.0004).

Western blot analysis showed that SERPINH1 (HSP47) protein levels were significantly higher in 5 matched GC tissues compared with the adjacent normal gastric mucosal tissues (Figure 5A). IHC analysis of 102 GC specimens showed that cytoplasmic expression of SERPINH1 was significantly higher in the GC tissues compared with the non-cancerous gastric mucosal tissues (Figure 5B–5E). As shown in Figure 5F, positive SERPINH1 protein staining was significantly higher in the GC tissues than in the adjacent normal gastric mucosal tissues (X^2^=8.485, P=0.004); high SERPINH1 protein levels were observed in 16 out of 48 normal adjacent gastric mucosal tissues (30%) compared with 60 out of 102 GC tissue samples (58.82%; Figure 5F).

Table 2 shows the association between SERPINH1 protein levels and the clinicopathological parameters in 102 GC patients. SERPINH1 protein expression was significantly higher in patients with advanced T (P=0.015), N (P<0.0001) and TNM (P<0.0001) stages, but showed no association with gender, age, tumor differentiation, tumor size, and M stage. Furthermore, GC patients with high SERPINH1 protein expression showed poorer OS than GC patients with low SERPINH1 expression, as analyzed by Kaplan–Meier survival analysis (Figure 5G). Multivariate Cox analysis demonstrated that high SERPINH1 protein expression was an independent prognostic factor (HR=4.054; 95% CI=1.30-12.54; P=0.016) in GC patients after adjustment for N and TNM stages (Table 3). Taken together, our data demonstrates that high SERPINH1 protein expression is associated with poorer survival rates in GC patients.

**Table 2:** Associations between SERPINE1 protein expression and clinicopathological features of 102 GC samples.

**Clinical features**		**SERPINE1 protein expression**		**P value**
		**Low expression(n=42)**	**High expression(n=60)**	
Gender	Female	12	19	
	Male	30	41	0.738
Age	<60	28	41	
	≥60	14	19	0.859
Differentiation	poor	30	48	
	well	12	12	0.315
Tumor size	<5cm	26	40	
	≥5cm	16	20	0.62
T stage	T1+T2	15	9	
	T3+T4	27	51	0.015
N stage	N0	24	11	
	N1	18	49	<0.0001
M stage	M0	41	53	
	M1	1	7	0.179
TNM stage	I+II	29	13	
	III+IV	13	47	<0.0001

**Table 3:** Univariate and multivariate Cox analyses of OS in 102 patients with GC.

**Clinical features**	**Univariate analysis**			**Multivariate analysis**		
	**HR**	**95%CI**	**P value**	**HR**	**95%CI**	**P value**
Gender	0.940	0.422-1.723	0.863			
Age	0.661	0.315-1.387	0.274			
G stage	0.819	0.337-1.989	0.659			
Tumor size	0.617	0.295-1.292	0.2			
T stage	1.517	0.677-3.401	0.311			
N stage	2.822	1.092-7.294	0.032	1.339	0.251-7.144	0.733
M stage	1.78	0.687-4.616	0.235			
TNM stage	2.518	1.097-5.781	0.029	1.18	0.277-5.026	0.823
SERPINE1 protein	4.954	1.734-14.151	0.003	4.054	1.305-12.544	0.016

### Enrichment analysis of genes co-expressing with SERPINH1 in the TCGA-STAD dataset

We analyzed the gene expression data from the TCGA-STAD dataset using the cBioPortal database and identified 87 genes that co-expressed with SERPINH1 (|Spearman r| > 0.5). Gene enrichment analysis using the FunRich software showed that these 87 co- expressed genes were involved in EMT, beta3 integrin cell surface interactions, integrin family cell surface interactions, beta1 integrin cell surface interactions, VEGFR3 signaling in lymphatic endothelium, integrins in angiogenesis and others (Supplementary Figure 1). Among these, EMT was the most significant signaling pathway that correlated with SERPINH1 expression (P<0.0001). These data suggest that SERPINH1 upregulation promotes GC metastasis via EMT.

### SERPINH1 regulates proliferation and survival of GC cells

**Figure 6:**
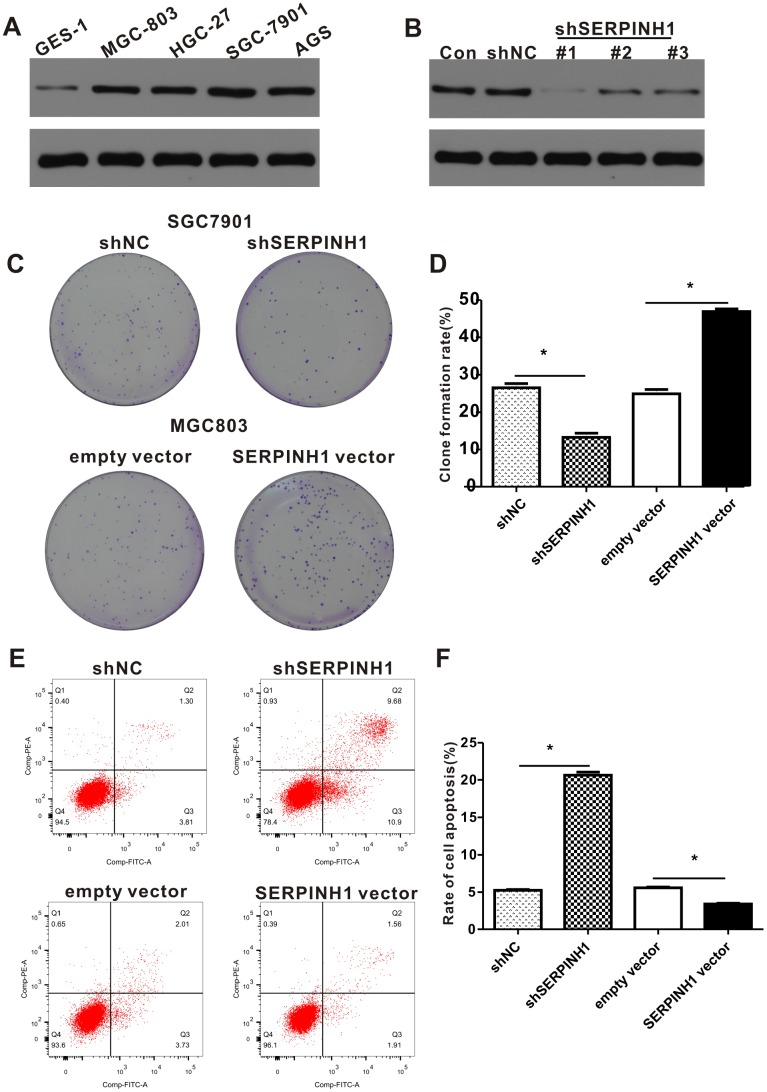
**SERPINH1 expression regulates the proliferation and survival of GC cell lines.** (**A**) Western blot analysis shows that SERPINH1 protein levels are higher in the four GC cell lines, HGC-27, AGS, MGC-803, SGC-7901, compared to the normal gastric mucosal cell line, GES-1. (**B**) Western blot analysis shows that SERPINH1 protein levels are significantly reduced in shSERPINH1 #1-transfected SGC-7901 cells compared with SGC-7901 cells transfected with shSERPINH1 #2, shSERPINH1 #2, and shNC. (**C**) Representative images of colonies in shSERPINH1#1-transfected SGC-7901 cells, SERPINH1-overexpression vector transfected MGC-803 cells, and their corresponding controls. (**D**) Histogram plots show the number of colonies in shSERPINH1#1-transfected SGC-7901 cells, SERPINH1-overexpression vector-transfected MGC-803 cells, and their corresponding controls. (**E**) Flow cytometry analysis shows that apoptotic rate is significantly higher in the shSERPINH1#1-transfected SGC-7901 cells and significantly lower in the SERPINH1-overexpression vector-transfected MGC-803 cells compared to their corresponding controls. (**F**) Histogram plot shows the percentage of apoptotic cells in shNC-, and shSERPINH1#1-transfected SGC-7901 cell cultures, as well as, empty vector and SERPINH1-overexpression vector-transfected MGC-803 cells.

Western blot analysis showed that SERPINH1 protein levels were significantly higher in four GC cell lines, namely, HGC-27, AGS, MGC-803, SGC-7901 compared with the normal gastric mucosal cell line, GES-1 (Figure 6A). Among the GC cell lines, SGC-7901 cells showed highest SERPINH1 expression and MGC-803 cells showed lowest SERPINH1 expression.

We investigated the role of SERPINH1 levels in GC by transfecting SERPINH1-specific shRNAs to downregulate SERPINH1 in SGC-7901 cells and transfecting SERPINH1-overexpression vector to enhance SERPINH1 levels in MGC-803 cells. Western blot analysis showed that SGC-7901 cells transfected with shSERPINH1 #1 showed significant SERPINH1 downregulation compared to SGC-7901 cells transfected with shSERPINH1 #2, #3, and shNC (Figure 6B). Therefore, we selected SGC-7901 cells transfected with shSERPINH1 #1 for further experiments. SERPINH1-silenced SGC-7901 cells showed significantly reduced number of colonies compared with the control (Figure 6C, 6D). Conversely, SERPINH1-overexpressing MGC-803 cells showed significantly higher number of colonies than the controls (Figure 6C, 6D). The SERPINH1-silenced SGC-7901 cells showed significantly higher rate of apoptosis than the control SGC-7901 cells, whereas, the SERPINH1-overexpressing MGC-803 cells showed significant reduction in apoptosis than the control MGC-803 cells (Figure 6E, 6F). These data demonstrate that SERPINH1 upregulation promotes proliferation and survival of GC cells.

### SERPINH1 regulates the migration and invasion of GC cells

**Figure 7:**
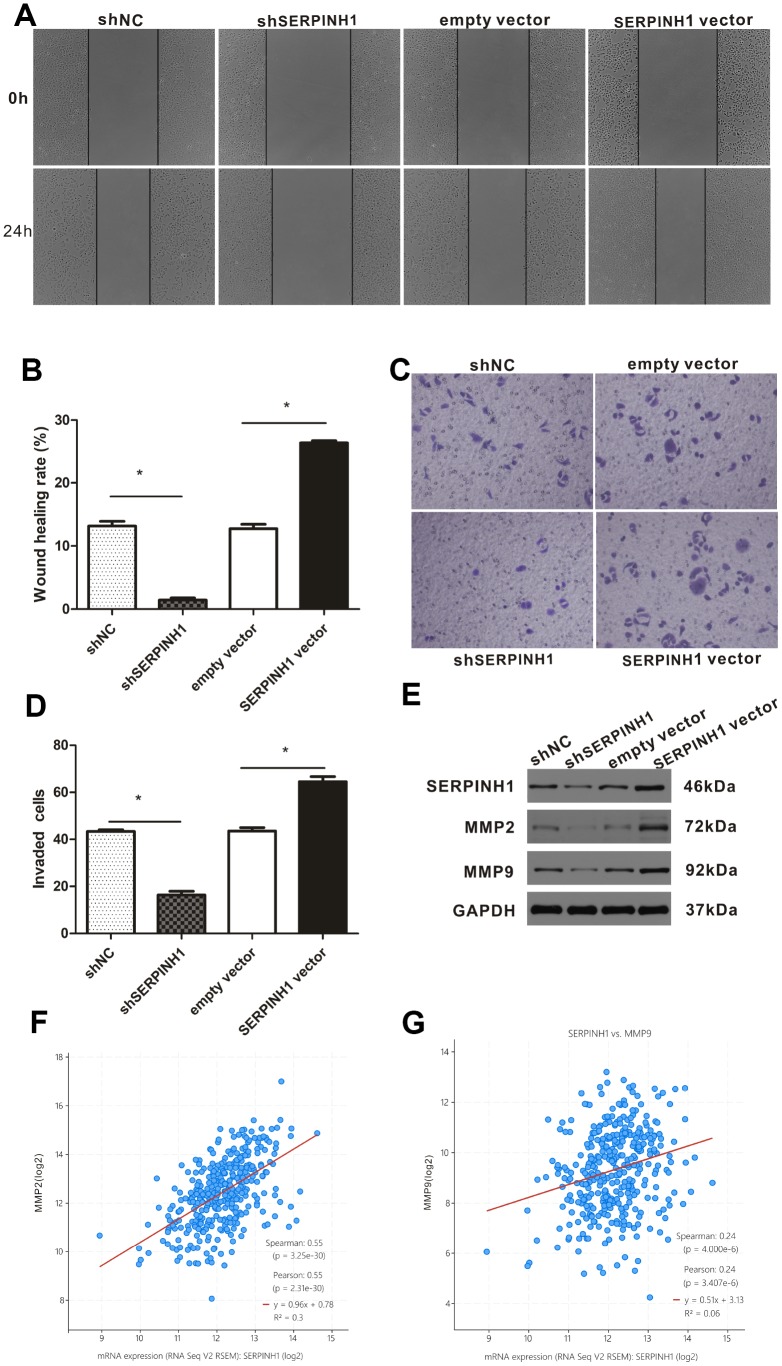
**SERPINH1 expression regulates *in vitro* migration and invasion of GC cells.** (**A**) Wound healing assay shows that the distance between wound edges was higher in SERPINH1-silenced SGC-7901 cells than in the control SGC-7901 cells at 24 h. Conversely, the distance between wound edges was significantly lower in the SERPINH1-overexpressing MGC-803 cells than in the control MGC-803 cells at 24 h. (**B**) Quantitative analysis of wound healing assay in the control and SERPINH1-silenced SGC-7901 cells, as well as control and SERPINH1-overexpressing MGC-803 cells. (**C**) Representative images show results of the Transwell migration assay, and (**D**) histogram plots show the number of migrating cells in the control and SERPINH1-silenced SGC-7901 cells, as well as control and SERPINH1-overexpressing MGC-803 cells. As shown, migration is reduced in SERPINH1-silenced SGC-7901 cells and increased in SERPINH1 overexpressed MGC-803 cells compared with the corresponding controls. (**E**) Western blot analysis shows that MMP2 and MMP9 protein levels are significantly reduced in the shSERPINH1-silenced SGC-7901 cells and increased in the SERPINH1-overexpressing MGC-803 cells compared with the corresponding controls. (**F**, **G**) Gene expression analysis shows that (**F**) MMP2 (r=0.55, P<0.0001) and (**G**) MMP9 (r=0.24, P<0.0001) mRNA levels positively correlate with SERPINH1 mRNA levels in GC patients from the TCGA-STAD dataset.

We performed the wound scratch assay to determine the effects of SERPINH1 silencing or overexpression on the migration of GC cells. The results showed reduced migration of the SERPINH1-silenced SGC-7901 cells and enhanced migration of the SERPINH1-overexpressing MGC-803 cells compared with their corresponding controls (Figure 7A, 7B). Transwell invasion assay showed enhanced invasiveness of the SERPINH1-overexpressing MGC-803 cells, and decreased invasiveness of the SERPINH1-silenced SGC-7901 cells compared with the corresponding controls (Figure 7C, 7D).

The expression of matrix metalloproteinases (MMP) correlates with the migration potential of cancer cells [18]. Therefore, we analyzed the expression of MMP2 and MMP9 in SERPINH1-overexpressing MGC-803, SERPINH1-silenced SGC-7901, and their corresponding controls. Western blot analysis showed that MMP2 and MMP9 levels were significantly reduced in the SERPINH1-silenced SGC-7901 cells, and significantly increased in the SERPINH1-overexpressing MGC-803 cells compared with their corresponding controls (Figure 7E). Furthermore, gene expression analysis of the TCGA-STAD dataset using the cBioPortal website showed that SERPINH1 mRNA expression significantly correlated with MMP2 (Pearson r=0.55, P<0.0001, Figure 7F) and MMP9 (Pearson r=0.24, P<0.0001, Figure 7G) mRNA levels. Overall, these data show that SERPINH1 expression regulates migration and invasion of GC cells.

### SERPINH1 regulates epithelial-mesenchymal transition of GC cells

**Figure 8:**
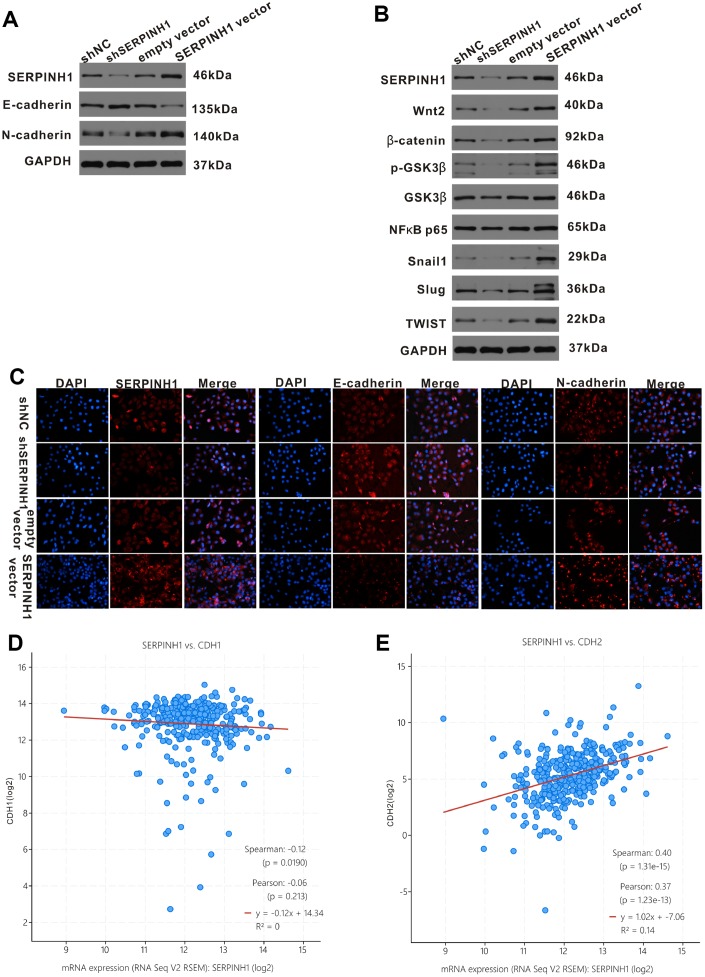
**SERPINH1 regulates EMT markers and Wnt/β-catenin signaling pathway in GC.** (**A**) Western blot analysis shows reduced N-cadherin and increased E-cadherin expression in SERPINH1-silenced SGC-7901 cells compared with controls. Conversely, SERPINH1-overexpressing MGC-803 cells show increased N-cadherin and reduced E-cadherin expression compared with the controls. (**B**) Western blot analysis shows increased levels of β-catenin, Wnt2, GSK-3β, p-GSK-3β, NF-κB p65, Snail1, Slug, and TWIST in the SERPINH1-overexpressing MGC-803 cells compared with the controls, whereas SERPINH1-silenced SGC-7901 cells show reduced levels of β-catenin, Wnt2, GSK-3β, p-GSK-3β, NF-κB p65, Snail1, Slug, and TWIST compared with the controls. (**C**) Immunofluorescence staining of E-cadherin, N-cadherin and SERPINH1 proteins in the control and SERPINH1-silenced SGC-7901 cells, as well as, control and SERPINH1-overexpressing MGC-803 cells. (**D**, **E**) Gene expression analysis of the TCGA-STAD dataset shows (**D**) negative correlation of CDH1 (r=-0.12, P=0.019) or E-cadherin mRNA levels and (**E**) positive association of CDH2 (r=0.40, P<0.0001) or N-cadherin mRNA levels with the SERPINH1 mRNA levels.

Next, we analyzed the relationship between SERPINH1 and EMT in GC cells. Western blot analysis showed significantly increased E-cadherin (epithelial marker) and decreased N-cadherin (mesenchymal marker) protein levels in SERPINH1-silenced SGC-7901 cells compared with control SGC-7901 cells; in contrast, SERPINH1-overexpressing MGC-803 cells showed increased N-cadherin and decreased E-cadherin expression compared with control MGC-803 cells (Figure 8A). We confirmed these data using immunofluorescence assays. The SERPINH1-overexpressing MGC-803 cells showed high SERPINH1, low E-cadherin and high N-cadherin protein levels; the SERPINH1-silenced SGC-7901 cells showed low SERPINH1, high E-cadherin and low N-cadherin protein levels (Figure 8C). Furthermore, gene expression analysis of the TCGA-STAD dataset using the cBioPortal website showed that SERPINH1 mRNA levels negatively correlated with levels of CDH1 or E-cadherin mRNA (r=-0.12, P=0.019; Figure 8D) and positively correlated with levels of CDH2 or N-cadherin mRNA (r=0.40, P<0.0001; Figure 8E).

### SERPINH1 regulates the Wnt/β-catenin pathway in GC cells

The Wnt/β-catenin signaling pathway plays a crucial role in regulating EMT in GC [16]. Therefore, we performed western blot analysis of key proteins involved in the Wnt/β-catenin signaling pathway, such as, β-catenin, Wnt2, GSK-3β, p-GSK-3β, NF-κB p65, Snail1, Slug, and TWIST) in SERPINH1-overexpressing and -silenced GC cells. The β-catenin, Wnt2, GSK-3β, p-GSK-3β, NF-κB P65, Snail1, Slug and TWIST protein levels were significantly reduced in the SERPINH1-silenced SGC-7901 cells, and significantly increased in the SERPINH1-overexpressing MGC-803 cells, compared with their corresponding controls (Figure 8B). Furthermore, gene expression analysis of the TCGA-STAD data using the cBioPortal website showed that SERPINH1 mRNA levels correlated positively with the mRNA levels of WNT2 (r=0.48), NF-κB (r=0.20), TWIST (r=0.52), SNAIL1 (r=0.67), and SNAIL2 (r=0.45), but showed no significant association with the mRNA levels of CTNNB1 or β-catenin and GSK3B (Supplementary Figure 2). These results suggest that SERPINH1 regulates the Wnt/β-catenin pathway in GC cells.

### Co1003 treatment reduces viability and migration of GC cells

**Figure 9:**
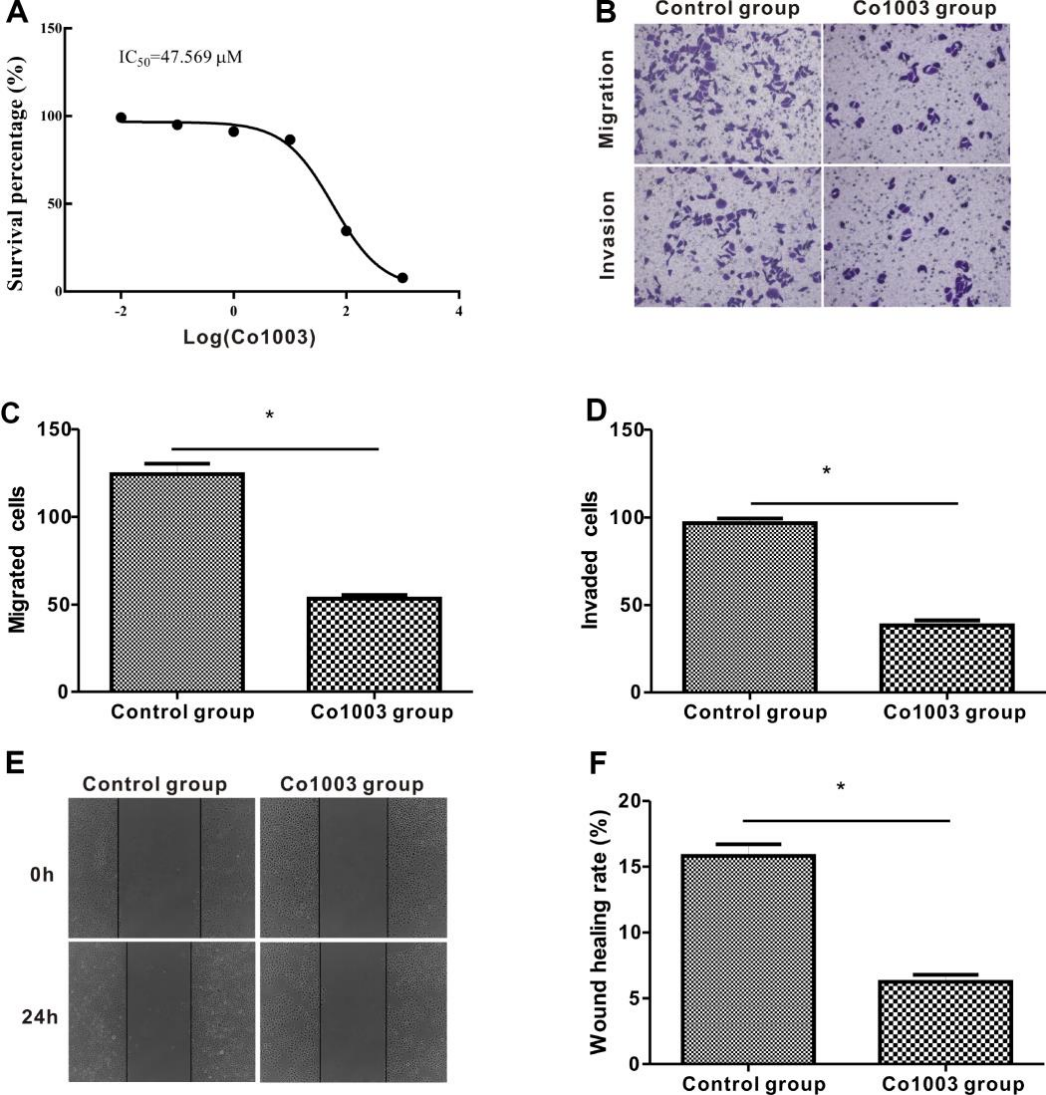
**Anti-tumor effects of CO1003 in GC cells.** (**A**) CCK-8 assay analysis shows concentration-dependent inhibition of proliferation of SGC-7901 GC cells by CO1003 (0, 0.01, 0.1, 1, 10, 100, 1000 μM). This curve was used to determine the IC_50_ concentration of CO1003, which is the concentration of CO1003 required to reduce proliferation of SGC-7901 cells by 50%. IC_50_ for CO1003 is 47.56 μM. (**B**) Transwell migration and invasion assay shows reduced migration and invasion of CO1003-treated SGC-7901 cells compared to the DMSO-treated GC cells. (**C**, **D**) Histogram plots show total number of (**C**) migrating and (**D**) invading DMSO- and CO1003-treated SGC-7901 cells. (**E**) Representative images of wound healing assay show that the distance between wound edges was significantly higher in Co1003-treated SGC-7901 cells compared with the DMS-treated SGC-7901 cells. (**F**) Wound healing assay shows lower wound healing rate because of reduced migration in CO1003-treated SGC-7901 cells compared with the DMSO-treated SGC-7901 cells.

Finally, we analyzed the anti-tumor effects of Col003, a small molecule inhibitor of the SERPINH1 protein. We incubated SGC-7901 cells in medium containing different concentrations of Co1003 (0, 0.01, 0.1, 1, 10, 100, 1000 μM) for 24h, and analyzed the cell viability using the CCK-8 assay. The results showed a concentration-dependent reduction of SGC-7901 cell viability by Col003 comparison with the DMSO-treated control SGC-7901 cells (Figure 9A). The IC_50_ of Col003 was 47.57 μM, and this concentration for further experiments. Transwell migration and invasion assay showed that Col003 significantly decreased migration and invasiveness of SGC-7901 cancer cells compared with DMSO-treated control SGC-7901 cells (Figure 9B–9D). Wound healing assay showed that inhibition of SERPINH1 protein with Col003 in SGC-7901 GC cells significantly reduced their migration compared with the control cells (Figure 9E, 9F). These results demonstrate that Col003 inhibits viability, migration, and invasion of GC cells, and is a promising therapeutic target for GC

## DISCUSSION

Epithelial mesenchymal transition (EMT) is an essential step for metastasis and correlates with poor prognosis in GC patients [19–22]. In this study, we demonstrate that SERPINH1 regulates EMT and metastasis in GC patients via Wnt/β-catenin signaling pathway.

SERPINH1 protein is essential for the correct folding of precursor collagen proteins (pro-collagen) in the endoplasmic reticulum [11]. SERPINH1 has been implicated in several collagen-related diseases, such as, brittle bone disease [23], keloid scars [24], and lung fibrosis [25–27]. Zhu et al showed that SERPINH1 regulates extracellular matrix (ECM) gene transcription and is involved in breast cancer progression [13]. SERPINH1 also correlates with the grade of glioma, and regulates proliferation, migration and invasion of glioma cell lines [14]. SERPINH1 protein expression was significantly higher in colorectal cancer patients with lymph node metastasis [15]. Zheng et al analyzed the GEO database and showed that SERPINH1 was one of the five hub genes in GC [28]. Cao et al analyzed plasma mRNA profiles of 56 GC patients and 14 healthy individuals in the Oncomine database and demonstrated high SERPINH1 mRNA levels in the GC samples [29]. In this study, we analyzed SERPINH1 mRNA expression in 888 GC and 318 normal gastric mucosal tissues from three public databases (Oncomine, TCGA, and GEO). Our results show that SERPINH1 mRNA levels are significantly higher in the GC tissues compared with the normal gastric mucosal tissues. GC patients with high SERPINH1 mRNA levels in the TCGA (N=415) and GEO (N=876) datasets are associated with poorer prognosis than those with low SERPINH1 mRNA levels. Moreover, IHC assay of 102 clinical GC samples shows that high SERPINH1 protein expression correlate with poorer prognosis in GC patients. These data suggest that SERPINH1 is a prognostic indicator in GC patients.

Wnt/β-catenin signaling pathway regulates EMT in several cancers, including GC; activation of the Wnt/β-catenin pathway increases β-catenin levels in the nucleus [30]. Another member of the Wnt/β-catenin pathway, GSK-3β, regulates the phosphorylation, degradation, and translocation of β-catenin [31]. The phosphorylation of β-catenin regulates its stability and determines the activity of the Wnt/β-catenin signaling pathway [32]. We analyzed the TCGA-STAD dataset and showed that SERPINH1 regulates the Wnt/β-catenin signaling pathway. Furthermore, SERPINH1 knockdown in SGC-7901 cells significantly decreases the levels of several members of the Wnt/β-catenin signaling pathway, namely, GSK-3β, β-catenin, Wnt2, TWIST, Snail, and Slug proteins. Conversely, overexpression of SERPINH1 increases the expression of Wnt signaling pathway proteins. Thus, our results demonstrate that SERPINH1 regulates the Wnt/β-catenin signaling pathway and modulates GC progression.

High SERPINH1 protein levels have also been reported in esophageal squamous cell carcinoma [33], cervical squamous cell carcinoma [12], and ulcerative colitis-associated carcinomas [15]. In scirrhous gastric cancer tissues and cells, SERPINH1 protein is localized in the ECM [34]. In our study, GC tissues show increased SERPINH1 protein expression compared to the normal gastric mucosal tissues. SERPINH1 protein binds to procollagen in the endoplasmic reticulum and helps in the folding and secretion of a mature functional collagen into the extracellular matrix [11]. The cancer-associated fibroblasts (CAFs) in the tumor stroma secrete significant amounts of collagen leading to higher collagen levels in the GC tissues than in the normal gastric tissues [34]. CAFs play a significant role in promoting EMT and GC progression [35–37]. This may be the reason why expression of SERPINH1 protein in GC tissues is higher than that in the normal gastric mucosal.

Inhibition of SERPINH1 protein might be a promising strategy not only for the treatment of fibrosis disease, but also for the treatment of GC. Shinya *et al* reported that Col003 is a small molecule competitive inhibitor of SERPINH1 protein [38]. We demonstrate that treatment of GC cells with Col003 significantly decreases their survival, migration and invasiveness. This suggests that Col003 is a potential therapeutic option for GC patients.

To the best of our knowledge, the present study is the first to explore the clinical significance and molecular function of SERPINH1 in GC. However, our study has several limitations. First, the study cohort of GC patients from our hospital was relatively small. Secondly, we could not analyze the relationship between SERPINH1 and PFS because of lack of PFS data. Moreover, we performed overexpression and silencing of SERPINH1 separately in distinct GC cell lines. These issues need to be addressed in future investigations.

In conclusion, our study demonstrates SERPINH1 regulates EMT and metastasis via the Wnt/β-catenin signaling pathway in GC. We demonstrate that SERPINH1 is a potential prognostic and diagnostic biomarker in GC. Finally, we demonstrate that SERPINH1 is a potential therapeutic target in GC patients.

## MATERIALS AND METHODS

### Clinical specimens

We obtained 102 GC and 48 adjacent noncancerous samples from the Department of Pathology in Renmin Hospital of Wuhan University. These samples were collected from patients that underwent surgery between 2015 and 2017. None of these GC patients received systematic radiotherapy or chemotherapy. We also obtained five pairs of fresh GC tissues and corresponding adjacent non-tumor gastric tissues during surgical resection from the Department of General Surgery in our hospital and stored the samples at −80 °C for western blot analysis. These samples were confirmed by two pathologists, Na Zhan and Zhi Zeng. We obtained written informed consent from all patients that participated in this study. This study was reviewed and approved by the Institutional Review Board of Renmin Hospital of Wuhan University.

### Gene expression data from public databases

We analyzed SERPINH1 mRNA levels in matched GC and normal gastric mucosal tissues using the Oncomine (http://www.oncomine.org), TCGA-STAD [https://xenabrowser.net/datapages/?cohort=GDC%20TCGA%20Stomach%20Cancer%20(STAD)] and GEO (GSE29272 and GSE54129 datasets) databases. The prognostic value of SERPINH1 mRNA levels was evaluated using the UCSC Xena (https://xenabrowser.net/heatmap/) database [39] and confirmed using the Kaplan-Meier Plotter (http://www.kmplot.com) database [17]. We analyzed genes that are co-expressed with SERPINH1 using the TCGA-STAD dataset (|spearman’s r|>0.5). They were analyzed using the cBioPortal (http://www.cbioportal.org/) database [40] and a functional enrichment network was constructed using the FunRich software (version 3.1.3).

### SERPINH1 protein inhibitor

Co1003 (HY-124817) was purchased from MedChem Express Co. Ltd. A stock solution of 10 mM Co1003 was prepared in dimethyl sulphoxide (DMSO) and stored at -20 °C.

### Immunohistochemistry

Immunohistochemistry (IHC) staining was performed as previously described [41]. Briefly, tissue sections were deparaffinized and rehydrated in graded ethanol solutions. Then, the deparaffinized tissue specimens were boiled in 10mM citrate buffer (pH 6.0) for 20 minutes for antigen retrieval. Subsequently, the endogenous peroxidase activity was blocked by incubating with 3% H_2_O_2_ solution. The tissue sections were then incubated with the anti-SERPINH1 protein (HSP47) antibody (1:300 dilution, ab109117, Abcam). The sections were developed using 3, 3’-diaminobenzidine (DAB) and counterstained with hematoxylin. The negative control sections were incubated in parallel with the immunoglobulin IgG (1:300) instead of the SERPINH1-specific antibody.

IHC staining was evaluated by two experienced pathologists using a semi-quantitative scoring system based on the intensity of staining and the percent of positively-stained cells. The staining intensity of specimens was scored as 0 (negative), 1 (weak), 2 (moderate) and 3 (strong). The positive expression of SERPINH1 protein was scored as 0 (0%), 1 (<25%), 2 (25%-50%), 3 (50%-75%), and 4 (>75%) based on the percentage of positive stained cells [42]. The final staining scores for all samples were obtained by multiplying the staining intensity and the positive SERPINH1 expression scores. The median score was used to categorize low or high SERPINH1 protein expression groups in order to determine prognosis of GC patients.

### Cell culture

The four human GC cell lines (HGC-27, AGS, MGC-803, SGC-7901) and the normal human gastric epithelium cell line (GES-1) were purchased from the China Center for Type Culture Collection (Wuhan, China). These cell lines were grown in DMEM/F-12 1: 1 medium (Hyclone, Logan, UT, USA) supplemented with 10% fetal bovine serum (FBS; Gibco, Thermofisher Scientific, Waltham, MA, USA) in a humidified chamber at 37°C and 5% CO_2_.

### CCK8 assay

Cell viability of GC cells was estimated using the CCK-8 assay. Experimental and control GC cells were seeded into 96-well plates at a density of 10^5^ cells per well for 24h. Then, 10 μL of CCK-8 reagent (C0038, Biyuntian biotechnology company) was added into each well and incubated for another 4 h at 37°C. The absorbance was measured at a wavelength of 450 nm. The concentration of Col003 required to reduce GC viability by 50% (IC_50_) was determined using the GraphPad Prism 5 software.

### Wound scratch assay

A scratch wound was made using a sterile 100 μl pipette tip in a monolayer of GC cells that were seeded in 6-well plates. The scratch wound was imaged at the 0 and 24 h time points using a phase contrast microscope. The mean distance between the two edges of cell free area was used to determine the wound healing or cell migration rate.

### Colony formation assay

GC cells (300 cells per well) stably transfected with control or shRNAs against SERPINH1 or control and SERPINH1 overexpression vector were cultured in 6-well plates at 37°C for 2 weeks. Then, the colonies were stained with 0.1% crystal violet. The colonies (>50 cells) were imaged and quantified under a light microscope.

### Transwell migration and invasion assay

We determined GC cell migration and invasion using the Transwell assay. For the invasion assay, we coated the polycarbonate membrane in the transwell chambers with Matrigel (Corning, USA). We transferred 2 x 10^4^ cells in serum-free medium into the top chamber and added medium with serum in the bottom chamber, and incubated at 37 °C for 24 h. Then, we removed the non-invading cells on the top side of the membrane by scrubbing, fixed the migrating or invading cells at the bottom side of the membrane with 4% paraformaldehyde (PFA), and stained with 0.1% crystal violet. The total number of cells was counted under the light microscope to determine the number of cells that migrated or invaded in each experimental group.

### Immunofluorescence staining

We fixed the cells by incubating in 4% PFA solution (AS1018, Aspen, Wuhan) for 30 min. Then, after blocking the fixed cells with 10% BSA (10735078001, Roche) at 37 °C for 30 min, they were incubated at 4 °C overnight with the following primary antibodies: anti-SERPINH1 (ab109117, Abcam); anti-E-cadherin (20874-1-AP, San Ying, Wuhan); and anti-N-cadherin (Abcam, Ab18203). Then, the cells were incubated with CY3- or FITC-conjugated goat anti–mouse or goat anti–rabbit IgG (AS-1111, Aspen, Wuhan) at 37 °C for 1 h. The cells were imaged and analyzed under a fluorescence microscope.

### Western blotting

We extracted total proteins from human GC tissues and cell lines using the RIPA lysis buffer (AS1004, ASPEN, Wuhan). Then, equal amounts of the protein lysates were separated on a 10% SDS PAGE. The resolved proteins were transferred onto PVDF membranes (IPVH00010, Millipore) and blocked with 5% non-fat milk in TBST for 2 h at room temperature. Then, the membrane was incubated overnight at 4°C with the following primary antibodies: anti- SERPINH1 (ab109117, Abcam), anti-Wnt2 (sc-514382, SANTA CRUZ), anti-β-Catenin (17565-1-AP, SanYing, Wuhan), anti-p-GSK3β (#5558, CST), anti-GSK3β (#12456, CST), anti-NFκB p65 (#8242, CST), anti-Snail1 (13099-1-AP, SanYing, Wuhan), anti-Slug (ab106077, Abcam), anti-TWIST (ab49254, Abcam), anti-MMP2(ab37150, Abcam), anti-MMP9 (ab76003, Abcam), anti-E-Cadherin (#3195, CST), anti-N-Cadherin (#4061, CST). Subsequently, the membranes were incubated with the horseradish peroxidase (HRP)-conjugated secondary antibody (AS1107, ASPEN). The blots were developed using the enhanced chemiluminescence system (AS1059, ASPEN) and the protein bands were imaged.

### Flow cytometry analysis of cellular apoptosis

We used the apoptosis detection kit (AO2001-02P-G, Sanjian, Tianjin) to estimate the apoptotic rate of different experimental groups of GC cells. We centrifuged 5×10^5^ cells for 5 min at 300xg and resuspended the cells in 300μl binding buffer. Then, the cells were incubated with 5 μL Annexin V-FITC for 10 min followed by 5 μL propidium iodide (PI) at 37°C in darkness for 5 min. Finally, the cells were analyzed by flow cytometry using the BD Aria III flow cytometer (BD Biosciences). The percentage of apoptotic cells in samples were determined by estimating the percentage of Annexin V ^+^ PI^+^ and Annexin V^+^ PI^-^ cells.

### SERPINH1-specific shRNAs and the SERPINH1-overexpression construct

We purchased SERPINH1 silencing shRNAs, shNC, empty vector control and SERPINH1-overexpressing vectors from Genepharma (Suzhou, China). The negative control and SERPINH1-targeting shRNAs were cloned into the lentiviral LV3-pGLV-h1-GFP-Puro vector. The SERPINH1 targeting shRNAs were: shSERPINH1#1 (SERPINH1-Homo-488), 5′-GCCTGGCCTTCAGCTTG TACC-3′; shSERPINH1#2 (SERPINH1-Homo-769), 5′-GCTGATGACTTCGTGCGCAGC-3′; and shSERPINH1#3 (SERPINH1-Homo-809), 5′-GCGAGCACTCCAAGATCAACT-3′. We cloned the PCR product containing the SERPINH1 open reading frame (NM_001207014.1) into the LV5-EF-1aF-GFP-Puro vector to overexpress SERPINH1 in GC cells. We then transfected the SGC-7901 cells with control and SERPINH1-silencing shRNAs, and MGC-803 cells with empty vector control and SERPINH1-overexpression vector using Lipofectamine 3000 (Invitrogen, USA) according to manufacturer’s instructions.

### Statistical analysis

Statistical analysis was performed using SPSS Statistics 20.0 (IBM SPSS, Chicago, IL) and GraphPad Prism 5 (GraphPad Software, CA, USA) softwares. The differences between two groups were analyzed using the Student’s t test. The association between SERPINH1 expression and the clinicopathological variables were estimated using the Pearson’s χ2 test. The prognostic significance of SERPINH1 was evaluated using the Kaplan–Meier plotter. Cox regression model was used to analyze the independent risk factors of survival. P < 0.05 was considered statistical significant.

## Supplementary Material

Supplementary Figures
