# Supplementary material for: SERPINH1 regulates EMT and gastric cancer metastasis via the Wnt/β-catenin signaling pathway
Source: Aging (Albany NY). 2020 Feb 24;12(4):3574–93. doi: 10.18632/aging.102831 (PMC7066881; doi:10.18632/aging.102831)
Supplement: Supplementary Figures [file aging-12-102831-s001..pdf]

SUPPLEMENTARY FIGURES

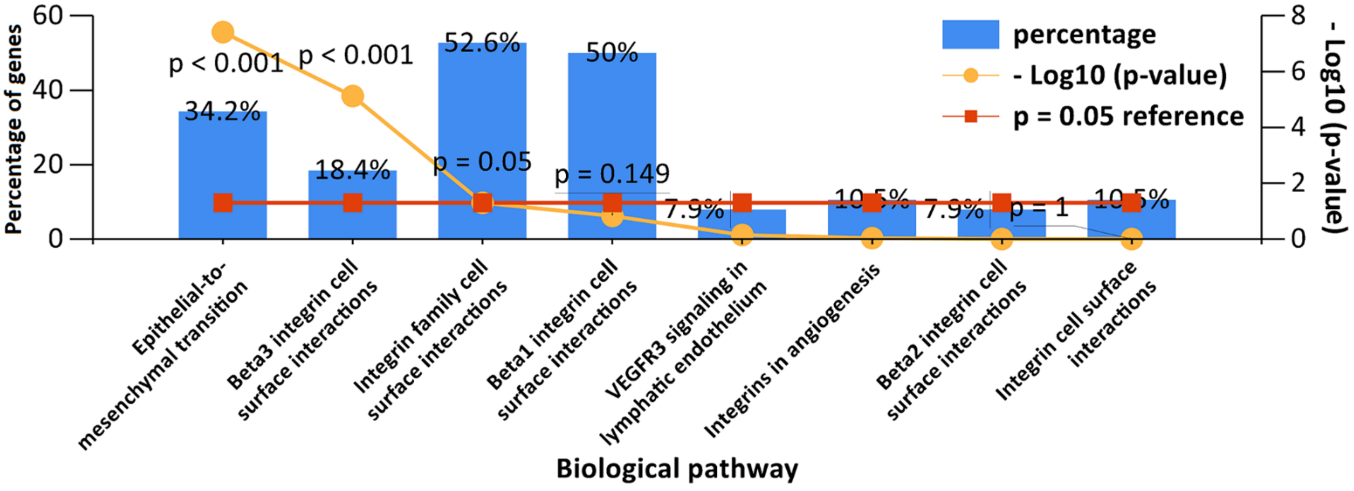

Supplementary Figure 1. Biological pathways that are differentially regulated in GC tissues by SERPINH1 based on the functional enrichment (FunRich) analysis of 87 genes that co-express with SERPINH1.

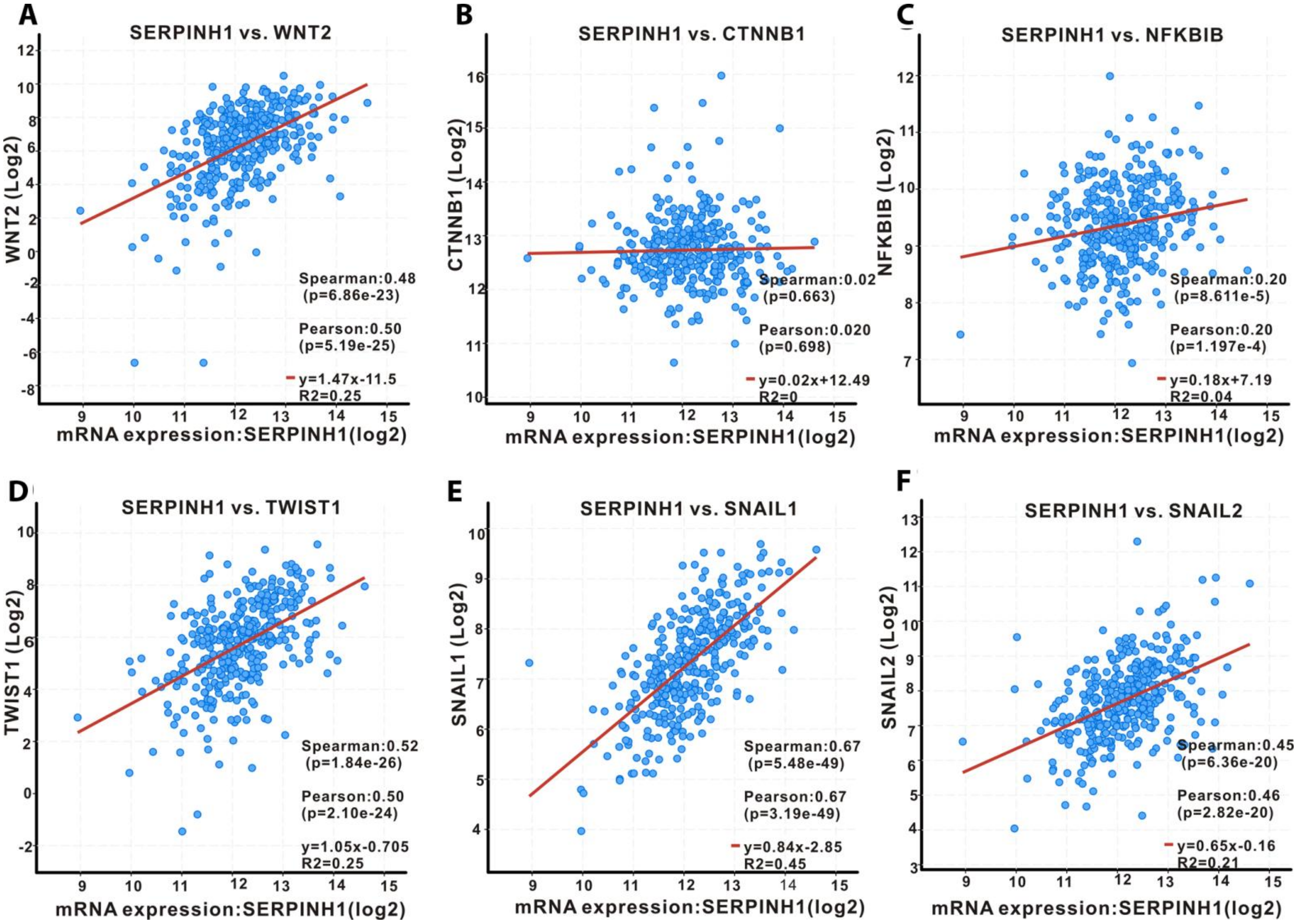

Supplementary Figure 2. Correlation analysis of SERPINH1 and Wnt/β-catenin pathway genes. (**A**) WNT2 and SERPINH1; (**B**) CTNNB1 or β-catenin and SERPINH1; (**C**) NFKBIB (NF-κB inhibitor B protein) and SERPINH1; (**D**) TWIST1 and SERPINH1; (**E**) SNAIL1 and SERPINH1; (**F**) SNAIL2 or Slug and SERPINH1.
